# Metacognitive beliefs mediate the relationship between anxiety sensitivity and traits of obsessive-compulsive symptoms

**DOI:** 10.1186/s40359-020-00412-6

**Published:** 2020-04-26

**Authors:** Roberto Gutierrez, Tulsi Hirani, Leo Curtis, Amanda K. Ludlow

**Affiliations:** Department of Psychology and Sports Sciences, University of Hertfordshire, Hatfield, AL10 9BB UK

**Keywords:** Metacognition, Anxiety sensitivity, Obsessive compulsive symptoms, State anxiety, Trait anxiety

## Abstract

**Background:**

Metacognition has been shown as a key contributor to Obsessive Compulsive Disorder as well as other anxiety-related disorders, yet its role in the development and maintenance of these disorders remains unclear. This study aims to investigate whether anxiety sensitivity traits are related to obsessive-compulsive symptoms in the general population and whether the relationship between anxiety sensitivity and obsessive-compulsive symptoms is mediated by metacognition.

**Methods:**

Non-clinical volunteers (*N* = 156, mean age: 23.97, 121 females) completed measures related to state/trait anxiety, anxiety sensitivity, obsessive compulsive symptoms and metacognition.

**Results:**

A direct relationship between anxiety sensitivity and obsessive-compulsive symptoms was established. Further analysis revealed that metacognition was the strongest mediator of this relationship, even when accounting for state and trait anxiety.

**Conclusions:**

Results suggest that the relationships between traits of anxiety sensitivity and obsessive-compulsive symptoms are partially attributable to the role of metacognition.

## Background

Obsessive Compulsive disorder (OCD) is classified by recurrent and intrusive thoughts (obsessions), as well as persistent behaviors (compulsions), which are created to combat the distress associated with obsessions [1, 2]. OCD is a condition that has a negative impact on the quality of life of the individual as well as their family [3, 4]. The prevalence of OCD in the general population may be higher than the estimated 1–2% previously reported [5, 6]. For example, results from an epidemiological study in the general population of six European countries showed a life-time prevalence of 13% [7]. Importantly, the prevalence of obsessive-compulsive symptoms (O-C) in the general population could be five times higher than in those reaching the threshold for a clinical diagnosis [5]. Moreover, O-C symptoms in childhood increase the chances of reaching a clinical diagnosis of OCD as an adult [7, 8], and highlights the need to further understand the development of O-C symptoms in the general population.

While more traditional cognitive accounts of OCD propose that symptoms arise from different types of dysfunctional beliefs, [9], recent metacognitive models have placed more emphasis on the way in which the intrusive thoughts are appraised in determining the symptoms of OCD [10]. Therefore, it is not only differences in the appraisal and beliefs about thoughts that are crucial to the development of (O-C) symptoms, but also the excessive attention and awareness of the thinking itself [11].

Metacognition is often referred to as the knowledge about our own thinking system, as well as factors and appraisals that affect our thinking [12]. In terms of mental health, it is proposed that metacognition can be a main factor in the development and maintenance of several psychological disorders. The Self-Regulatory Executive Function model (S-REF) [13, 14] was put forward to address the relationship between these metacognitive beliefs (e.g., “I cannot control my thoughts”) and affective disorders [15]. The S-REF model proposed that metacognitive beliefs heighten self-focused attention, whilst simultaneously reducing the ability to process information that would challenge any dysfunctional belief. According to this model, one’s pattern of responses, known as the Cognitive-Attentional Syndrome (CAS), leads to a tendency to process negative information through perseverative thinking (e.g., worry), threat monitoring, avoidance, and thought suppression. The CAS is driven by beliefs and knowledge about one’s thoughts and cognitive processes (e.g., memory, attention), that can involve both, positive metacognitive beliefs about the usefulness of engaging in aspects of CAS (e.g., “worry helps me to focus”), and negative metacognitive beliefs about thoughts and feelings. Importantly, it is these negative metacognitive beliefs that have been found to be particularly influential in enhancing the CAS, through their feelings of loss of control and threatening interpretations of mental events [14].

Studies showed metacognitive beliefs as an underlying contributor to a range of affective disorders characterized by rumination and worry, including OCD [11], and metacognitive therapy showed to be an effective and time efficient treatment for OCD [16]. Moreover, the relationship between metacognition and O-C symptoms was found not only on individuals reaching a clinical diagnosis of OCD but but also is present in the general population [17].

Anxiety is a multifaceted construct often refer to as feelings of worry, fear and unease that can be caused by internal and external threats [18]. State anxiety is a temporary emotional state that includes feelings of apprehension, worry and nervousness [19], whereas trait anxiety refers to a relatively stable individual disposition to evaluate environmental events as threatening [20]. The state-trait-anxiety model suggests that state anxiety reactions depends to some extent of the level of trait anxiety [21, 22]. Previous research found that state and trait anxiety are strongly associated to O-C symptoms in clinical and non-clinical populations [23, 24], and it is suggested that trait anxiety and anxiety sensitivity are closely related constructs [25].

An additional cognitive risk factor affecting O-C symptoms is anxiety sensitivity, a trait-like characteristic that can predispose an individual to fear anxiety related sensations, in particular to physical, psychological and social concerns [26]. Anxiety sensitivity differs from trait anxiety, in specifically fearing the anxiety symptoms rather than being fearful of a range of stressors. Anxiety sensitivity has been implicated in the cause as well as the maintenance of OCD symptoms, with elevated anxiety sensitivity associated with difficulty experiencing and tolerating anxiety related sensations [27]. Moreover, in a study involving 87 treatment seeking adults with OCD, Storch and colleagues found that elevated anxiety sensitivity accounted for the severity of O-C symptoms [28], and was also associated with increased functional impairment.

Higher levels of anxiety sensitivity were shown to predict worse treatment outcome of Cognitive Behavior Therapy (CBT) in OCD [29]. This may be due to some obsessions directly relating to anxiety sensitivity, but also those individuals scoring higher in this trait show reluctance to engage in anxiety-provoking components of CBT. Importantly, the relationship between anxiety sensitivity and O-C symptoms has been demonstrated in a range of studies, including clinical and non-clinical populations [25, 30–32], and even when controlling for comorbid anxiety and depression diagnoses [33]. However, the role of metacognition in this relationship has yet to be explored.

Although previous findings suggest a causal and maintenance role of metacognitive beliefs on OCD [34, 35], few studies have considered the existence of an intermediary role of metacognition [36]. This role is important to address, as whilst being overly attentive towards one’s own process of thinking is a characteristic associated to patients with OCD as well as those who show a proneness to worry [37], many researchers have failed to establish a direct effect of anxiety on OCD and vice-versa [38]. Furthermore, using a non-clinical sample of university students, Irak and Tosun [24] found that metacognition was a full mediator of the relationship between O-C symptoms and trait anxiety, suggesting metacognition to be partially accountable for this relationship rather than just an additional contributor to O-C symptoms.

In the current study we aimed to address the interplay of metacognitive beliefs, anxiety sensitivity and O-C symptoms using a non-clinical sample. Given the role metacognitive beliefs play in one’s coping mechanisms and regulating emotions, previous levels of anxiety sensitivity could become a symptom in the presence of these metacognitive beliefs. We firstly hypothesized that metacognitive beliefs and anxiety sensitivity would both be independent predictors of O-C symptoms. Secondly, we predicted that metacognitive beliefs would mediate the relationship between anxiety sensitivity and O-C symptoms. Finally, we expected that metacognitive beliefs would remain a mediator of this relationship even when accounting for the effect of state and trait anxiety.

## Method

### Participants

One hundred and sixty-six university students from different disciplines at the University of Hertfordshire (United Kingdom) took part in the study on voluntary basis. The responses of 10 participants were discarded because they did not fully complete at least one of the questionnaires presented, leaving a total of 156 participants (35 males, 121 females). The mean age of the participants was 23.97 years old (*SD* = 7.61, *Min*. = 18, *Max*. = 51). The majority of participants identified themselves as White (97, 62.18%), 40 identified themselves as Asian (25.64%), 12 as Black (7.69%), and 7 as other (4.48%). All participants were recruited from the university campus.

### Materials

The *Anxiety Sensitivity Index* [39]. The inventory consists of 16 items that assesses concerns regarding anxiety related sensations in three different dimensions of anxiety sensitivity: physical (“It scares me when I am short of breath”), cognitive (“It scares me when I am unable to keep my mind on a task”), and social (“It is important to me not to appear nervous”). The scale uses a 5 point scale from 0 (“very little”) to 4 (“very much”), with higher scores indicating higher anxiety. The anxiety sensitivity index had a Cronbach’s alpha = .91 and has been shown to have good internal consistency, reliability and validity [40], and has been used previously in similar samples [41, 42]. A total score was calculated following the instructions of the scale to create an single score of anxiety sensitivity per participant.

The *State and Trait Anxiety Inventory* is a 40-item questionnaire, which measures the severity of anxiety symptoms [43]. It distinguishes between state anxiety as a temporary condition experienced in specific situations (“I am worried”), and trait anxiety that is seen as a general tendency to perceive situations as threatening (“I worry too much over something that really doesn’t matter”). The state and trait anxiety inventory is scored in a 4 point scale from 1 (“almost never” / “not at all”) to 4 (“almost always” / “very much so”). The inventory (*α* = .86) was scored according to the instructions of the scale in order to obtain 2 indexes per participant, one for state anxiety and one for trait anxiety.

The *Metacognition Questionnaire* is a 65-item scale used to measure the beliefs individuals have about their thinking [37]. It consists of five factors: positive worry beliefs (“Worrying helps me to avoid problems in the future”), beliefs about uncontrollability and danger (“My worrying is dangerous for me”), cognitive competence (“I have little confidence in my memory for words and names”), general negative beliefs (“If I did not control a worrying thought, and then it happened, it would be my fault”), and cognitive self-consciousness (“I think a lot about my thoughts”). The metacognition questionnaire is scored in a 4 point scale from 1 (“do not agree”) to 4 (“agree very much”) and had a Chronbach’s alpha = .88. The metacognition questionnaire has been used extensively in non-clinical samples such as smokers and students [44, 45]. A total score was calculated in order to create an index of metacognition per participant.

The *Obsessive-Compulsive Inventory* is a 42-item self-report questionnaire, assessing the severity of various obsessions and compulsions of OCD in adults [46]. The obsessive-compulsive inventory consists of 7 subscales that include washing (“I wash my hands more often or longer than necessary”), checking (“I go back to places to make sure that I have not harmed anyone”), doubting (“Even when I do something very carefully I feel that it is not quite right”), ordering (“I get upset if others have changed the way I have arranged my things”), obsessing (“I find it difficult to control my thoughts”), hoarding (“I collect things I don’t need”), and neutralising (“I feel I have to repeat certain numbers”). This inventory is scored on a 4 point Likert scale, from 1 (“not at all”) to 4 (“extremely”) and had a Chronbach’s alpha = .88. A total score was calculated in order to create one score per participant related to O-C symptoms.

### Procedure

An opportunity sample of university students took part in the study. Participants were firstly presented with information about the study and given the opportunity to ask questions about it. After giving consent, each participant provided demographic information, and then were presented with the four questionnaires in a counterbalanced order to answer at their own time. When the participant finished he or she was thanked and debriefed. All the materials and procedures followed the ethical guidelines and procedures outlined by the American Psychological Association and were checked and approved by the University of Hertfordshire ethics committee (Number:14149216).

## Results

Data was screened for missing values, outliers and assumptions of statistical analysis prior to any analysis [47]. All variables had acceptable values for skewness and kurtosis and no extreme outliers. Bivariate correlations of the scores of all the computed indexes revealed positive and significant associations between them (Table 1). The strongest association was between the indexes of state and trait anxiety, followed by the association between anxiety sensitivity, metacognition and O-C symptoms.

**Table 1:** Mean, Standard deviations and bivariate correlations of Anxiety Sensitivity, Metacognition, O-C symptoms, State anxiety and Trait anxiety

	Mean	SD	Min	Max	Skewness	Kurtosis	(1)	(2)	(3)	(4)
(1) Anxiety Sensitivity	24.73	13.28	0	60	.46	−.30	–			
(2) Metacognition	125.66	28.55	73	215	.50	−.01	.64	–		
(3) O-C Symptoms	33.71	23.26	0	116	1.06	.76	.48	.64	–	
(4) Trait Anxiety	44.40	10.98	20	76	.02	−.50	.51	.55	.58	–
(5) State Anxiety	40.33	11.05	20	69	.42	−.41	.46	.55	.56	.75

*Note*: All correlations had *p* values <.001

Our first prediction was that metacognition and anxiety (state and trait) would be significant predictors of O-C symptoms. Consistent with our expectations all variables were associated to O-C symptoms in significant and positive correlations (all *ps* < .001) [24, 30, 33].

In order to investigate the relative importance of each of the computed index on the prediction of O-C symptoms all the indexes were used as simultaneous predictors of O-C symptoms in a multivariate regression analysis. Results revealed that metacognition was the best predictor of O-C symptoms (*β* = .41, *p* < .001), followed by trait anxiety (*β* = .21, *p* < .05), state anxiety (*β* = .16, *p* = .08), and anxiety sensitivity (*β* = .04, *p* = .63). The overall model resulted significant, *R*^2^ = .49, *F*(4, 151) = 36.18, *p* < .001). Although previous research indicated that anxiety sensitivity is a good predictor of O-C symptoms [24], the inclusion of the other variables reduced its effect to a non-significant level.

Our second prediction was that the relationship between anxiety sensitivity and O-C symptoms would be mediated by both anxiety levels (state and trait) and metacognition levels. In order to test this prediction we performed a single-step parallel mediational analysis using the PROCESS macros and instructions provided by Hayes [48]. This analysis used a bootstrapping method and 10,000 repetitions, simultaneously entering the 3 mediators. The age and gender of the participants were entered as covariates in the analysis. All the results reported are standardized coefficients and 95% confidence intervals.

**Fig. 1:**
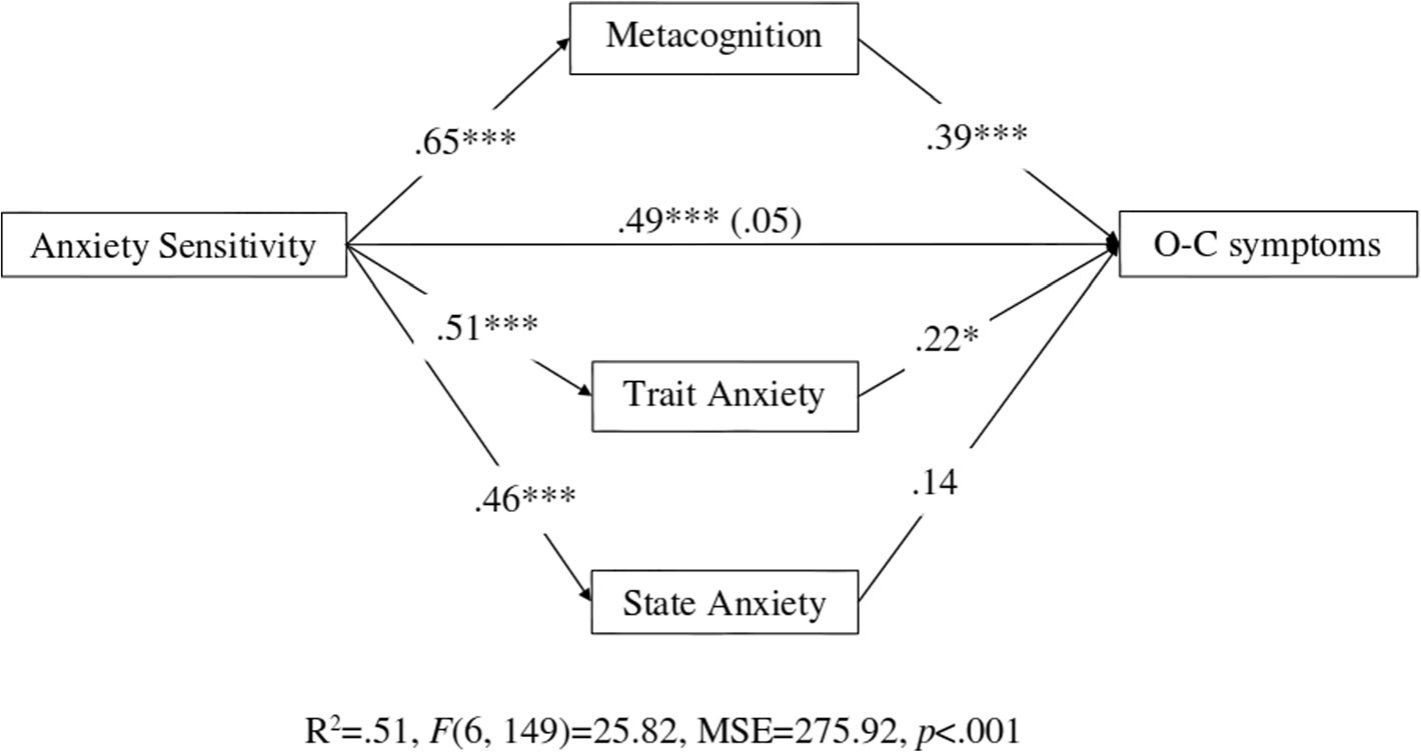
Metacognition, state and trait anxiety as mediators of the relationship between anxiety sensitivity and obsessive-compulsive symptoms

Results revealed that the total effect of anxiety sensitivity on O-C symptoms was significant (.49, se = .12, *t* = 7.01, *p* < .001, LCI = .61, UCI = 1.09), but was reduced to a non-significant level once the mediators were included (.05, se = .14, *t* = .70, *p* = .48, LCI = -.17, UCI = .37). Of the mediators analysed (Fig. 1), metacognition had the strongest indirect effect on O-C symptoms (.39, se = .07, LCI = .19, UCI = .45, *p* < .001). Trait anxiety also had a significant indirect effect (.22, se = .20, LCI = .08, UCI = .85, *p* < .05), whereas the indirect effect of state anxiety was not significant (.14, se = .19, LCI = -.07, UCI = .67, *p* = .11). Results of the contrast between these mediators revealed that metacognition was significantly different than state anxiety (.19, se = .09, LCI = −.37, UCI = -.02; but not significantly different to trait anxiety (.14, se = .09, LCI = -.34, UCI = .03). The contrast between trait anxiety and state anxiety was not significant (−.05, se = .09, LCI = -.23, UCI = .13). Finally, gender had no significant effects on any of the variables (all *ps* > .12), and age only had a significant effect on O-C symptoms (*β* = − .14, *p* < .05), suggesting that O-C symptoms diminish with age (all other *p* > .20). These results suggest that the effect of anxiety sensitivity on O-C symptoms is fully mediated by the levels of metacognition and trait anxiety to a lesser extent^1^.

## Discussion

We predicted that levels of O-C symptoms would be affected by levels of anxiety sensitivity, trait anxiety, state anxiety, and levels of metacognition. The results supported this prediction as all the variables studied showed strong and significant correlations. In addition, the present study also confirmed a direct relationship between anxiety sensitivity and O-C symptoms in a non-clinical population, with higher levels of anxiety sensitivity predicting higher levels of O-C symptoms [25]. Anxiety sensitivity was suggested as a cognitive risk factor for OCD; for example, those with high anxiety sensitivity consider unpleasant body sensations as a sign of illness [29]. In addition, anxiety sensitivity has also been highlighted as a cognitive risk factor for anxiety [30]. These results extend previous findings showing a strong relationship between levels of anxiety sensitivity and levels of O-C symptoms, and that this relationship is present in a non-clinical sample [32].

Our second prediction was that the relationship between anxiety sensitivity and O-C symptoms would be mediated by metacognition. There is support for this prediction as previous findings showed that metacognitive beliefs predict a variety of disorders such as health anxiety [49], OCD [35], and depression [50]. In addition, previous results showed that metacognition mediated the relationship between trait anxiety and O-C symptoms [24]. We extended such findings showing that anxiety sensitivity is a significant predictor of trait anxiety as well as state anxiety, and that metacognition is a stronger mediator when compared to trait and state anxiety on the relationship between anxiety sensitivity and O-C symptoms. Although O-C symptoms were correlated with trait anxiety and state anxiety, their relationship with O-C symptoms was diminished when metacognition was taken into consideration.

This pattern of results suggest that anxiety related sensations can increase and maintain their influence on O-C symptoms via metacognitive beliefs. The findings of this study also highlight the importance of metacognition in the relationship between anxiety sensitivity and O-C symptoms over and above the effect of state and trait anxiety. Our analyses showed that all variables were significant predictors of O-C symptoms when taken independently but when comparing these variables in a parallel analysis, metacognition was the strongest mediator of O-C symptoms.

There are some limitations in the current study that should be noted. As expected, there was a high correlation between state and trait anxiety and O-C symptoms, complicating their individual contribution to the relationship between anxiety sensitivity and O-C symptoms. As highlighted by Backstead [51], the correlation between these variables complicates the interpretation of each of these relationships. Furthermore, while metacognition was identified as the best mediator in the relationship between anxiety sensitivity and O-C symptoms, trait anxiety was also a mediator in this relationship. This association was highlighted by the individual correlations between the three variables tested as mediators (metacognition, state anxiety and trait anxiety), but the parallel mediation results allowed for the comparison of the 3 effects simultaneously.

All assessments used in this study relied on self-report measures over one testing session. While these tools represent a good clinical standard, self-report symptoms have been shown to fluctuate over time [52]. For example, some individuals who initially scored high in assessing compulsive behaviors of OCD were found to score within the normal range upon a second administration of a self-report measure [53]. Therefore, to confirm and extend our preliminary findings, self-report measures should be taken over several different time periods, and using the most recent versions of the measures. For example, the use of the original measures of the ASI and OCI were selected for use in the current study due to their good psychometric properties, but the decision not to use the most recent versions have raised significant limitation to note. While the ASI remains the most adopted measure of anxiety sensitivity [54], the 18-item Anxiety Sensitivity Index-3 has been shown to improve the basic psychometric criteria of the original scales, with the three subscales accounting for 76% of the variance compared to 60% of the original scale [26]. The original OCI is also a widely used and validated self-report measures, however when the scale was created, hoarding symptoms were coded under obsessive-compulsive disorder. The DSM-5 [1] saw the introduction of Hoarding Disorder (HD) as an independent diagnosis and thus the symptoms included in the original OCI now cross over two separate diagnostic categories. While this is also true of latest version the OCI-R, separate clinical cut offs have been shown to be effective in assessing likely diagnosis of both HD and OCD [55], making it a more appropriate tool to assess the symptoms of OCD. Furthermore, to highlight the pivotal role of metacognition on the relationship between anxiety sensitivity and O-C symptoms these findings should be corroborated and extended in samples with clinical levels of OCD and/or anxiety disorder. Despite some of these limitations, this is the first study to illustrate that metacognition has a mediating effect on the relationship between anxiety sensitivity and O-C symptoms in the general population. In addition, the characteristics of our sample was consistent with other research using non-clinical populations, showing O-C symptoms to diminish with age [56].

As OCD and O-C symptoms exists on a continuum, the relationships of these variables in non-clinical samples may be consistent with levels present in clinical populations [57]. These results suggest that treatment of O-C symptoms could be implemented using metacognition. For example, a recent intervention showed that thoughts about bodily sensations mediated the relationship between anxiety sensitivity and O-C symptoms [58]; and metacognitive therapy has already shown to be successful in some treatments of OCD [59]. Moreover, recent results suggest that metacognitive therapy is effective on treating depression and anxiety [60], as well as health anxiety [57].

## Data Availability

The datasets generated and analyzed during the current study will be available from the author upon reasonable request.

## Abbreviations

OCD: Obsessive Compulsive disorder
O-C: obsessive-compulsive symptoms
S-REF: Self-Regulatory Executive Function model
CAS: Cognitive-Attentional Syndrome
CBT: Cognitive Behavior Therapy
LCI: Lower Confidence Interval
UCI: Upper Confidence Interval

## Footnotes

1 We performed three additional mediation analyses in order to check for potential independent effects of the anxiety sensitivity index subscales. The total score of the anxiety sensitivity index was replaced by the subscale score as the main predictor (cognitive, physical and social), with metacognition, state anxiety and trait anxiety as parallel mediators. Results were almost identical, with metacognition having the strongest mediation effect on the 3 subscales and reducing the direct effect of each of the scores on O-C symptoms to a non-significant level.
